# eHealth profile of patients with diabetes

**DOI:** 10.3389/fpubh.2023.1240879

**Published:** 2023-08-16

**Authors:** Mathieu Jendly, Valérie Santschi, Stefano Tancredi, Isabelle Konzelmann, Leila Raboud, Arnaud Chiolero

**Affiliations:** ^1^Population Health Laboratory (#PopHealthLab), University of Fribourg, Fribourg, Switzerland; ^2^La Source, School of Nursing Sciences, HES-SO University of Applied Sciences and Arts Western Switzerland, Lausanne, Switzerland; ^3^Observatoire Valaisan de la Santé (OVS), Sion, Switzerland; ^4^School of Population and Global Health, McGill University, Montreal, QC, Canada; ^5^Institute of Primary Health Care (BIHAM), University of Bern, Bern, Switzerland

**Keywords:** eHealth, mHealth, diabetes, quality of care, digital health technology, self-management, equity

## Abstract

**Background:**

Digital health technology can be useful to improve the health of patients with diabetes and to support patient-centered care and self-management. In this cross-sectional study, we described the eHealth profile of patients with diabetes, based on their use of digital health technology, and its association with sociodemographic characteristics.

**Methods:**

We used data from the “Qualité Diabète Valais” cohort study, conducted in one region of Switzerland (Canton Valais) since 2019. Participants with type 1 or type 2 diabetes completed questionnaires on sociodemographic characteristics and on the use of digital health technology. We defined eHealth profiles based on three features, i.e., ownership or use of (1) internet-connected devices (smartphone, tablet, or computer), (2) mHealth applications, and (3) connected health tools (activity sensor, smart weight scale, or connected blood glucose meter). We assessed the association between sociodemographic characteristics and participants’ eHealth profiles using stratified analyses and logistic regression models.

**Results:**

Some 398 participants (38% women) with a mean age of 65 years (min: 25, max: 92) were included. The vast majority (94%) were Swiss citizens or bi-national and 68% were economically inactive; 14% had a primary level education, 51% a secondary level, and 32% a tertiary level. Some 75% of participants had type 2 diabetes. Some 90% of the participants owned internet-connected devices, 43% used mHealth applications, and 44% owned a connected health tool. Older age and a lower educational level were associated with lower odds of all features of the eHealth profile. To a lesser extent, having type 2 diabetes or not being a Swiss citizen were also associated with a lower use of digital health technology. There was no association with sex.

**Conclusion:**

While most participants owned internet-connected devices, only about half of them used mHealth applications or owned connected health tools. Older participants and those with a lower educational level were less likely to use digital health technology. eHealth implementation strategies need to consider these sociodemographic patterns among patients with diabetes.

## 1. Background

Diabetes is a major burden on the healthcare system and can have a major impact on patients’ quality of life (1). The risk of developing diabetes and its complications increases with age and lower levels of education (2). Within a patient-centered, evidence-based, and data-informed framework (3), improving the quality of care of patients with diabetes requires a multidisciplinary team of healthcare professionals, including physicians, pharmacists, or nurses (4, 5). Digital health technology, also known as eHealth, could help improve diabetes management by facilitating data exchange between patients and healthcare professionals, thus improving care practice and coordination. Within the field of eHealth, a distinction is made between mHealth (which refers to mobile health via applications on smartphones) and connected health tools (to measure and analyze health data) (6). For example, applications to monitor diet or exercise are considered as mHealth and digital blood glucose meters are connected health tools.

While there is no consensus on the effectiveness of eHealth in improving health outcomes, mounting evidence suggests that it could help, including among patients with diabetes (7). Hence, studies have shown that the use of digital health technology and mobile devices could help improve diabetes management, e.g., to reach lower level of glycated hemoglobin (HbA1c) or blood pressure (8, 9) as well as to improve psychosocial and physical well-being (10). It could also enable patient empowerment by allowing them to play an active role in the management of their disease (11). eHealth also has a positive impact on medication adherence (12) and in the management of polypharmacy (13), which is a major challenge among patients with chronic diseases like diabetes (14, 15).

Several factors however constrain eHealth tools implementation. Not all healthcare professionals are comfortable with digital health technology (16). Hence, they often have a poor digital health literacy and are not yet sufficiently trained to use these tools. The absence of strong evidence on their beneficial use, the ever-changing market of eHealth tools, and their cost are also limiting factors (17, 18). Furthermore, several barriers at the patient level hinder the use of eHealth tools, including socioeconomic and demographic factors (17). Indeed, there is a socioeconomic divide in digital health, with patients of lower socioeconomic status having lower digital literacy and limited access to digital health tools (19, 20). A scoping review on the factors influencing the digital divide identified age, ethnicity, and education, together with health status and eHealth literacy, as factors influencing the use of digital health technologies (21). A recent example that highlighted the lack of equity in digital health was the COVID-19 pandemic, where populations with the lowest socioeconomic status were less likely to integrate digital health innovations into their daily lives or usual care (22). Socio-patterns of eHealth use need to be better understood if policymakers are to design equitable strategies to promote digital health (23), including among patients with diabetes.

Our aim was, therefore, to evaluate the eHealth profile of patients with diabetes, based on their use of digital health technology, and assess its association with sociodemographic characteristics.

## 2. Participants and methods

### 2.1. Study design

We used data from a prospective cohort study (“Qualité Diabète Valais”) conducted by the Observatoire Valaisan de la Santé (OVS^1^) which aims to evaluate the quality of care and the quality of life of patients with diabetes in the canton of Valais, Switzerland (24, 25). For the current study, we performed a cross-sectional analysis of the data collected at baseline. Ethical approval was obtained to conduct the cohort study (ethics committee “Commission cantonale d’éthique de la recherche sur l’être humain CER-VD,” file number 2019–01668).

### 2.2. Participants

Participants were recruited in collaboration with several institutions and healthcare professionals in the Canton of Valais. Eligible patients were informed about the objectives of the study and the possibility of participating in the cohort. Volunteers were enrolled after a telephone interview with the OVS between December 2019 and December 2022. During the interview, detailed information about the study was provided and inclusion and exclusion criteria were reviewed. To be included in the study, participants had to meet the following inclusion criteria: (1) be 25 years or older; (2) have a physician-confirmed diagnosis of type 1 or 2 diabetes; (3) residency in Valais (Switzerland); (4) be able to take informed decisions. Exclusion criteria were: (1) gestational diabetes; (2) corticoid-induced diabetes; (3) severe cognitive impairment or other illness that prevented understanding the content of the information sheet; (4) insufficient knowledge of the French or German language (24). All participants signed an informed consent form.

### 2.3. Data collection and measurement

At baseline, participants completed a self-administered questionnaire to assess their sociodemographic characteristics, health status, quality of care, use of the health care system, health behaviors, quality of life and use of digital health technology.

For the current analysis, we used the following sociodemographic data and participants’ characteristics: age, sex, citizenship (Swiss or bi-national, non-Swiss), type of diabetes (type 1 or 2), marital status (single, in a relationship), household type (living alone, living in a couple), occupational status (active, inactive) and highest education level attained (primary, secondary, tertiary). Eight participants reported not knowing their type of diabetes. Type 2 diabetes is much more frequent in the population and type 1 diabetes requires greater patient involvement, making them more aware of the diagnosis. Furthermore, these participants were diagnosed at an old age. They were therefore assigned to the “type 2″ group. To describe the participants’ eHealth profile, we used the following information on digital health technology use: (1) ownership of an internet-connected device (smartphone, tablet, or computer); (2) use of a mHealth application (such as diet or exercise applications) and reasons for not using mHealth applications; (3) ownership of a connected health tool (activity sensor, smart weight scale, or connected blood glucose meter). We did not have information on the type of mHealth applications used by participants.

### 2.4. Statistical analysis

We presented descriptive statistics of the participants’ characteristics and their eHealth profile. We examined the association between participants’ characteristics and participants’ eHealth profile using stratified analyses and logistic regression models. Two models fitted to estimate the odds ratios: a model 1 unadjusted and a model 2 adjusted for age, sex, citizenship, type of diabetes, marital status, household type, occupational status, and highest education level attained. Statistical analyses were performed using Stata 17 software (Stata Corp, TX, 2021).

## 3. Results

The characteristics of the 398 participants recruited between December 2019 and December 2022 are shown in Table 1. The mean age was 65 years (min: 25, max: 92) and 38% were women. The majority were Swiss citizens or bi-national (94%) and economically inactive (68%). Some 14% had a primary level education, 51% a secondary level, and 32% a tertiary level. Most of the participants (75%) had type 2 diabetes.

**Table 1:** Characteristics of participants (*N* = 398).

Characteristics		%
Age [year]	Mean (Min; Max)	64.8 (25; 92)
	25–59 yr	31
	60–74 yr	49
	75 yr. or more	20
	Missing	<1
Sex	Women	38
	Men	62
Citizenship	Swiss and bi-national	94
	Other	6
	Missing	<1
Marital status	Single	44
	In a relationship	55
	Missing	1
Household type	Living single	34
	Living in a couple	56
	Other	4
	Missing	7
Occupational status	Active	31
	Inactive	68
	Missing	2
Highest education level attained	Primary	14
	Secondary	51
	Tertiary	32
	Missing	3
Type of diabetes	Type 1	25
	Type 2	75

Descriptive statistics of the participants’ eHealth profile are shown in Table 2. Some 90% of the participants owned an internet-connected device (smartphone, tablet, or computer), and 43% reported using mHealth. When asked about the reasons for not using mHealth, the most common responses were that they did not need it (45%), that it was too complicated (34%), that they did not find the application relevant to their health problem (14%). Only 2% said that it was too expensive. Some 44% of participants owned a connected health tool, the most common being connected blood glucose meters (31%) and activity sensors (25%).

**Table 2:** eHealth profile of participants (*N* = 398).

eHealth profile		%
1. Ownership of an internet-connected device	Yes	90
	No	9
	Missing	1
2. Use of mHealth	Yes	43
	No	47
	Does not know what it is	4
	Missing	6
3. Ownership of a connected health tool	Activity sensor	25
	Smart weight scale	6
	Connected blood glucose meter	31
	Any of the above	44
	Does not know what it is	5
	No ownership	44
	Missing	8

More than one answer was possible.

**Figure 1:**
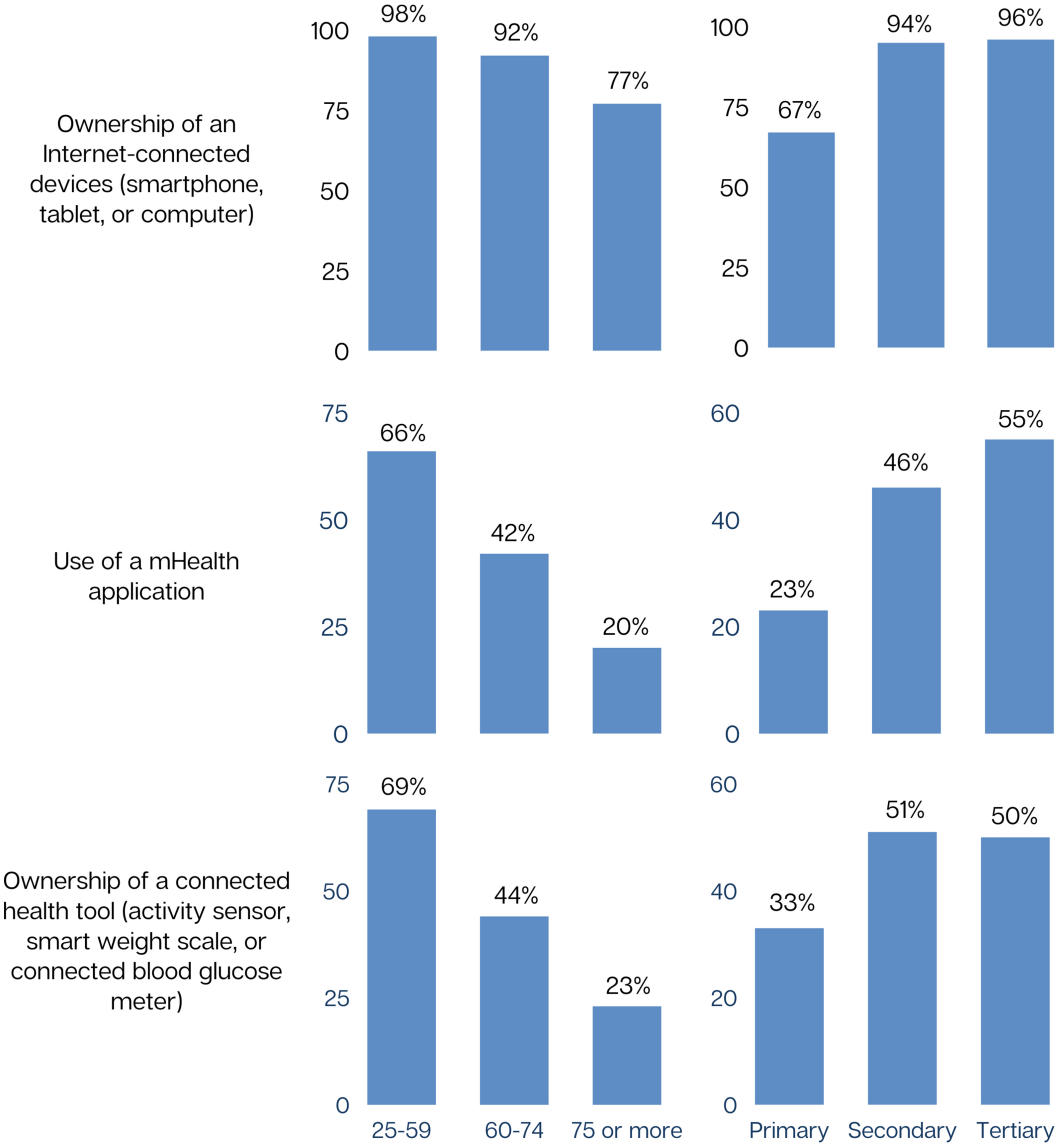
eHealth profile by age and education (*N* = 398).

The eHealth profile of participants according to their characteristics is shown in Supplementary Table S1 and Figure 1. All features of eHealth profile were related to age and to level of education. The higher the age and the lower the educational level, the less likely participants were to own internet-connected devices, to use mHealth applications, and to own connected health tools (Figure 1).

After adjusting for participants’ characteristics, age and education remained associated with owning an internet-connected device (Table 3), using mHealth applications (Table 4), and owning connected health tools (Table 5). Use of mHealth and ownership of connected health tools were less frequent among Non-Swiss and type 2 diabetes patients. Features of the eHealth profile were not associated with sex, marital status, occupational status, or household type.

**Table 3:** Association between participants’ characteristics and ownership of an internet-connected device (*N* = 398).

		Model 1		Model 2	
Characteristics		OR	95% CI	OR	95% CI
Age [year]	25–59 yr	1		1	
	60–74 yr	0.18	0.04–0.82	0.46	0.08–2.64
	75 yr. or more	0.05	0.01–0.24	0.16	0.03–0.98
Sex	Women	1		1	
	Men	1.93	0.97–3.84	0.84	0.33–2.14
Citizenship	Swiss and bi-national	1		1	
	Other	0.65	0.18–2.30	0.53	0.12–2.39
Marital status	Single	1		1	
	In a relationship	1.87	0.93–3.74	1.05	0.36–3.06
Household type	Living single	1		1	
	Living in a couple	1.73	0.82–3.67	1.44	0.36–5.75
	Other	1.64	0.20–14.43	1.64	0.16–16.51
Occupational status	Active	1		1	
	Inactive	0.06	0.01–0.42	0.19	0.02–1.66
Highest education level attained	Primary	1		1	
	Secondary	7.11	3.21–15.75	5.11	1.83–14.22
	Tertiary	11.97	4.16–34.43	9.81	2.80–34.34
Type of diabetes	Type 1	1		1	
	Type 2	0.58	0.23–1.44	0.95	0.31–2.88

Model 1: analyses are unadjusted; model 2: analyses are adjusted for participants’ characteristics. OR, odds ratio; CI, confidence interval.

**Table 4:** Association between participants’ characteristics and use of mHealth (*N* = 398).

		Model 1		Model 2	
Characteristics		OR	95% CI	OR	95% CI
Age [year]	25–59 yr	1		1	
	60–74 yr	0.37	0.23–0.60	0.44	0.23–0.85
	75 yr. or more	0.13	0.06–0.26	0.19	0.08–0.45
Sex	Women	1		1	
	Men	1.07	0.71–1.63	0.95	0.56–1.61
Citizenship	Swiss and bi-national	1		1	
	Other	0.49	0.19–1.31	0.36	0.12–1.05
Marital status	Single	1		1	
	In a relationship	1.38	0.91–2.08	1.27	0.71–2.28
Household type	Living single	1		1	
	Living in a couple	1.65	1.06–2.59	1.44	0.68–3.08
	Other	0.99	0.31–3.22	0.96	0.27–3.48
Occupational status	Active	1		1	
	Inactive	0.35	0.22–0.54	0.89	0.48–1.65
Highest education level attained	Primary	1		1	
	Secondary	2.95	1.38–6.20	1.85	0.75–4.60
	Tertiary	4.13	1.88–9.09	3.28	1.27–8.48
Type of diabetes	Type 1	1		1	
	Type 2	0.29	0.17–0.47	0.40	0.23–0.70

Model 1: analyses are unadjusted; model 2: analyses are adjusted for participants’ characteristics. OR, odds ratio; CI, confidence interval.

**Table 5:** Association between participants’ characteristics and ownership of a connected health tool (*N* = 398).

		Model 1		Model 2	
Characteristics		OR	95% CI	OR	95% CI
Age [year]	25–59 yr	1		1	
	60–74 yr	0.35	0.21–0.57	0.37	0.19–0.73
	75 yr. or more	0.13	0.07–0.26	0.16	0.07–0.39
Sex	Women	1		1	
	Men	1.29	0.84–1.97	1.30	0.76–2.23
Citizenship	Swiss and bi-national	1		1	
	Other	0.48	0.18–1.29	0.28	0.09–0.86
Marital status	Single	1		1	
	In a relationship	1.24	0.82–1.88	0.69	0.38–1.27
Household type	Living single	1		1	
	Living in a couple	1.78	1.13–2.81	2.02	0.90–4.54
	Other	2.52	0.78–8.16	2.48	0.68–9.07
Occupational status	Active	1		1	
	Inactive	0.31	0.20–0.50	0.76	0.41–1.44
Highest education level attained	Primary	1		1	
	Secondary	2.11	1.07–4.17	1.17	0.50–2.73
	Tertiary	2.00	0.98–4.09	1.45	0.59–3.57
Type of diabetes	Type 1	1		1	
	Type 2	0.29	0.17–0.48	0.39	0.22–0.70

Model 1: analyses are unadjusted; model 2: analyses are adjusted for participants’ characteristics. OR, odds ratio; CI, confidence interval.

## 4. Discussion

The aim of this study was to describe the eHealth profile of patients with diabetes in one region of Switzerland, and its relationship with sociodemographic factors. We found that most participants owned an internet-connected device (smartphone, computer, or tablet) and that about half of them used mHealth applications and owned connected health tools, especially connected blood glucose meters and activity sensors. Older age and a lower educational level were associated with lower odds of all features of the eHealth profile. To a lesser extent, having type 2 diabetes was also associated with a lower use of digital health technology. This could be due to differences in treatment approaches for type 1 and type 2 diabetes, as type 1 diabetes is treated with insulin and requires strict glucose monitoring before insulin administration, and type 2 diabetes is often treated with metformin, without the need to measure glucose levels beforehand. Not being a Swiss or bi-national citizen was also associated with a lower use of digital health technology. There was no association between sex and the features of the eHealth profile.

A survey conducted in Switzerland in 2022 found that around half of the Swiss population used digital health tools (mHealth and connected health tools combined). Greater use was associated with younger age, higher education, higher income, and greater digital health literacy (26). Our findings are also consistent with some other studies on eHealth use in patients with diabetes or other chronic diseases. For example, a 2019 literature review investigating socio-demographic factors influencing eHealth use in patients with chronic diseases found that “higher age and lower income, lower education, living alone, and living in rural areas were […] associated with lower eHealth use” (27). In another study conducted in Australia, several factors were associated with a lower likelihood of access to eHealth: higher age, lower education, low digital literacy, low socioeconomic status, and living in remote areas (28). In addition, one study found that the same factors-older age, ethnic minority, and lower education-were associated with less readiness to use areas of eHealth such as teleconsultation and digital prescribing. Patients with diabetes were also less ready for eHealth than the general population (29).

While this study focuses only on personal characteristics of patients associated with eHealth use, the acceptability of eHealth to healthcare professionals is also another important determinant of eHealth use. In an umbrella review, it was found that positive attitudes of healthcare professionals toward eHealth (such as beliefs that the new systems would benefit patients, interest in the technologies, usefulness, and motivation in working with the systems) increase its acceptance and implementation, while negative attitudes (beliefs that electronic systems would disrupt the delivery of care; doubts that these systems can improve patient care, clinical outcomes or the quality of medical practices; and distrust in the systems as well as a more general staff resistance to change) decrease it (30). The current study shows that the use of eHealth by patients with diabetes is quite high, and that should drive health professionals to give more interest in the use of this technology in usual clinical activity.

This study has a few limitations. First, the sample size was relatively small. Second, we recruited participants on a voluntary basis, implying that our sample is only partly representative of the population of patients with diabetes. For example, the proportion of participants with type 1 diabetes (25%) was much higher than in the general population, where type 1 diabetes accounts for approximately 5–10% of all diagnosed cases of diabetes (31). Moreover, recruiting only voluntary participants might have introduced a “healthy user bias,” participants probably being better informed and having a higher health and digital health literacy compared to usual patients with diabetes. Third, information bias is also possible because the information collected by the questionnaire was self-reported. Fourth, we had no information on why patients were using eHealth tools. Finally, participants were relatively old and we could not accurately assess the eHealth profile of young participants with diabetes. More research is needed to further investigate the use of eHealth in younger patients with diabetes.

Our results suggest that older patients of relatively low educational level, who are the patients with the highest diabetes related morbidity and mortality rates, are also the least likely to use eHealth. If the benefits of eHealth are confirmed, it means that policy for its implementation should target these groups to have their access to eHealth strengthened. The Organization for Economic Co-operation and Development (OECD) has published a report suggesting that in order to implement eHealth in clinical practice, governments need to meet three objectives: increase the confidence of healthcare workers and patients in the benefits of digital transformation, increase the knowledge and skills needed to use digital health technologies effectively, and adapt the organization of healthcare services and the legal and financial operating frameworks (32).

In conclusion, while most patients with diabetes owned internet-connected devices such as smartphones, tablets, or computers, only about half of them used mHealth applications or owned connected health tools. The oldest participants and those with lower educational levels were the least likely to use eHealth, despite being the groups that could benefit the most from it.

## Data availability statement

Datasets are available upon request to the corresponding author.

## Ethics statement

The studies involving humans were approved by ethics committee “Commission cantonale d’éthique de la recherche sur l’être humain CER-VD,” file number 2019-01668. The studies were conducted in accordance with the local legislation and institutional requirements. Written informed consent for participation in this study was provided by the participants.

## Author contributions

AC, IK, and LR designed the “Qualité Diabète” study. MJ, VS, and ST wrote the protocol for the current analysis. IK and LR recruited participants and managed data necessary for this study. MJ analyzed the data under the guidance of ST and drafted the manuscript with contributions of VS, ST, and AC. All authors revised and approved the final version of the manuscript before submission.

## Funding

The “Qualité Diabète” project is financed by the canton of Valais and the Valais diabetes associations via the “Action Diabète” project supported by Promotion Santé Suisse. There is no specific funding for this analysis.

## Conflict of interest

The authors declare that the research was conducted in the absence of any commercial or financial relationships that could be construed as a potential conflict of interest.

## Publisher’s note

All claims expressed in this article are solely those of the authors and do not necessarily represent those of their affiliated organizations, or those of the publisher, the editors and the reviewers. Any product that may be evaluated in this article, or claim that may be made by its manufacturer, is not guaranteed or endorsed by the publisher.

## Footnotes

^1^ www.ovs.ch
