# Supplementary material for: eHealth profile of patients with diabetes
Source: Front Public Health. 2023 Aug 16;11:1240879. doi: 10.3389/fpubh.2023.1240879 (PMC10466783; doi:10.3389/fpubh.2023.1240879)
Supplement: Supplementary file 1 [file Table_1.docx]

Supplementary Material

eHealth profile of patients with diabetes

# Supplementary Table

| *Characteristics* |  | Ownership of an internet-connected device | mHealth use | Ownership of a connected health tool |
| --- | --- | --- | --- | --- |
|  | % | 90 | 43 | 44 |
| Age [year] | 25-59 yr | 98 | 66 | 69 |
|  | 60-74 yr | 92 | 42 | 44 |
|  | 75 yr or more | 77 | 20 | 23 |
| Sex | Women | 87 | 44 | 44 |
|  | Men | 93 | 46 | 50 |
| Citizenship | Swiss and bi-national | 91 | 46 | 49 |
|  | Other | 87 | 30 | 32 |
| Marital status | Single | 88 | 41 | 45 |
|  | In a relationship | 93 | 49 | 50 |
| Household type | Living single | 89 | 39 | 39 |
|  | Living in a couple | 93 | 51 | 53 |
|  | Other | 93 | 39 | 62 |
| Occupational status | Active | 99 | 63 | 68 |
|  | Inactive | 87 | 37 | 39 |
| Highest education level attained | Primary | 67 | 23 | 33 |
|  | Secondary | 94 | 46 | 51 |
|  | Tertiary | 96 | 55 | 50 |
| Type of diabetes | Type 1 | 94 | 68 | 70 |
|  | Type 2 | 90 | 38 | 40 |

**Supplementary Table 1.** eHealth profile according to participants’ characteristics (N = 398).
